# Europium(III) Complex-Functionalized SiO_2_@mTiO_2_ Nanospheres for Al^3+^-Modulated Multicolor Emission

**DOI:** 10.3390/nano11112886

**Published:** 2021-10-28

**Authors:** Chao Bai, Shi He, Huai-Ming Hu, Hui Zeng, Feng Zou, Ji-Jiang Wang

**Affiliations:** 1 Key Laboratory of New Energy & New Functional Materials, Shaanxi Key Laboratory of Chemical Reaction Engineering, College of Chemistry and Chemical Engineering, Yan’an University, Yan’an 716000, China; baichao_chem@163.com; 2 Key Laboratory of Synthetic and Natural Functional Molecule of the Ministry of Education, College of Chemistry and Materials Science, Northwest University, Xi’an 710127, China; hans5113@163.com (S.H.); 17863522138@163.com (H.Z.); 201931739@stumail.nwu.edu (F.Z.)

**Keywords:** lanthanide hybrid materials, SiO_2_@mTiO_2_ core–shell nanosphere, bipyridine derivative, multicolor luminescence

## Abstract

A europium(III) hybrid material Eu(tta)_3_bpdc-SiO_2_@mTiO_2_ (Htta = 2-thenoyltrifluoroacetone, H_2_bpdc = 2,2′-bipyridine-3,3′-dicarboxylic acid) was successfully designed and synthesized by the covalent grafting complex Eu(tta)_3_bpdc to SiO_2_@mTiO_2_ core–shell nanosphere. The FT-IR, PXRD, XPS, TEM, HRTEM, SAED, TGA and PL were performed to characterize these materials. The results indicate that core–shell nanosphere structure and anatase crystallites of SiO_2_@mTiO_2_ are retained well after grafting the europium complex. Hybrid material Eu(tta)_3_bpdc-SiO_2_@mTiO_2_ displays uniform nanosphere structure, bright red color and long lifetime, which can serve as a multicolor emission material modulated by using Al^3+^ ions via the cation exchange approach under a single-wavelength excitation. To the best of our knowledge, this work is the first multicolor emissive sensor for Al^3+^ ions based on the lanthanide hybrid material.

## 1. Introduction

Lanthanide-based luminescent hybrid materials have sharp emission bands, high color purity, long luminescent lifetime and high quantum efficiency. They can also act as excellent candidates for multicolor luminescent materials, and further are applied in many fields, such as bio-imaging and bioanalytical detection [1,2], luminescent probes and sensors [3,4,5], tunable luminescence [6,7,8], and optoelectronic devices and displays [9,10,11]. In the past years, color-tunable lanthanide-based materials usually control the stoichiometric ratios of doped ions in the same material to realize multicolor emission (containing white-light emission) [12,13,14,15]. Recently, Li et al. have achieved some progress in the field of single-component lanthanide-based hybrid materials with multicolor emission [16,17]. They declared that full-color emission, particularly white emission colors of europium-based hybrid materials, can be facilely tuned by using only a kind of cation or anion due to the cation exchange process or interaction between ligands and anions. However, to date, single-component lanthanide hybrid materials with multicolor emission by using the cation exchange approach remain a challenge.

Aluminum is the third most abundant metal in the Earth’s crust [18], it has been widely used in the fields of aluminum containers, cooking tools, food additives, water treatment, and aluminum-based pharmaceuticals. According to the standard of WHO, the average human intake of aluminum is about 3.0~10.0 mg/day and weekly dietary intake is 7.0 mg/kg body weight [19,20,21]. The excessive intake of aluminum can lead to the production of neurotoxic issues in humans such as Parkinson’s or Alzheimer’s disease [18,22]. Therefore, the selective detection and control of Al^3+^ ions in biological systems are essential. Fluorescence-based probes as a kind of simple and rapid responsive method have drawn a lot of interest for sensing applications due to these methods being low-cost, very sensitive, selective and visualizable while offering rapid response times and real-time monitoring. To date, several fluorescent Al^3+^ sensors such as organic compounds (Schiff base [23], phenylhydrazine carboxamide [24], coumarin-rhodamine derivative [25], amino derivative [26]), Cd-MOF [27] and europium complexes [28,29] have been reported. Noticeably, lanthanide-based hybrid materials have received considerable attention as a sensor for detecting Zn^2+^ [16] and Cu^2+^ [30], due to their novel and unique properties, such as photostability, adjustable pore size, large surface area, as well as easy recyclability.

On the basis of above consideration, we designed and fabricated a europium(III) hybrid material based on SiO_2_@mTiO_2_ core–shell nanosphere (namely, Eu(tta)_3_bpdc-SiO_2_@mTiO_2_) with covalent grafting complex Eu(tta)_3_bpdc (Htta = 2-thenoyltrifluoroacetone, H_2_bpdc = 2,2′-bipyridine-3,3′-dicarboxylic acid). The resulting core–shell hybrid material Eu(tta)_3_bpdc-SiO_2_@mTiO_2_ shows multicolor emission based on sensing of Al^3+^ ions by the cation exchange process. The emission color changed from red to pink and blue based on luminescence intensity changes by modulating only the concentration of Al^3+^ ions in an ethanol solution of hybrid material Eu(tta)_3_bpdc-SiO_2_@mTiO_2_. To the best of our knowledge, this work is the first multicolor emissive sensor for Al^3+^ ions based on the lanthanide hybrid material.

## 2. Experiment Section

### 2.1. Materials

1,10-Phenanthroline monohydrate (phen, AR, Aladdin, Shanghai, China), 2-thenoyltrifluoroacetone (Htta, AR, J&K, Beijing, China), tetraethyl orthosilicate (TEOS, Si(OC_2_H_5_)_4_, AR, J&K, Beijing, China), hexadecylamine (HDA, AR, J&K, Beijing, China), titanium(IV) isopropoxide (Tip, AR, J&K, Beijing, China) were purchased from a commercial source without any further purification. Hydrated EuCl_3_ was prepared by dissolving europium(III) oxide (99.99%) in concentrated hydrochloric acid.

### 2.2. Preparation of H_2_bpdc Ligand

To deionized water (350 mL), phen (0.04 mol, 8.00 g), NaOH (0.08 mol, 3.20 g) and KMnO_4_ (0.12 mol, 19.09 g) were added. The mixture was heated to 100~105 °C and stirred for 5 h, and then cooled to room temperature. The filtrate was obtained by filtration and then concentrated to 100 mL and adjusted the pH of the filtrate to 2.0 by HCl (aq, conc.). The resulting white precipitate was filtered off, recrystallized with methanol and dried in vacuo to give colorless needle-like crystals, yield: 8.2 g, 85%. Elemental Anal. Calc. for C_12_H_8_N_2_O_4_ (244.05): C, 59.02; H, 3.30; N, 11.47%. Found: C, 59.12; H, 3.15; N, 11.54%.

### 2.3. Preparation of Eu(tta)_3_(H_2_O)_2_

The complex Eu(tta)_3_(H_2_O)_2_ was synthesized according to the reported literature [31]. An appropriate amount of 1.0 M sodium hydroxide solution was added to an Htta ethanol solution to adjust pH = 7.0 under stirring at room temperature. Subsequently, a hydrated EuCl_3_ ethanol solution was dropwise added into the above mixture and kept the molar ratio of Eu^III^/Htta equal to 1/3. An appropriate amount of deionized water was added to the mixture and heated at 65 °C for 5 h, then cooled to room temperature. The yellow precipitates were obtained by filtration, then washed with cold ethanol and deionized water three times.

### 2.4. Preparation of SiO_2_@mTiO_2_ Core–Shell Nanospheres

The SiO_2_@mTiO_2_ core–shell nanospheres were synthesized according to the reported literature [32,33]. (i) SiO_2_ nanospheres were prepared by a modified Stöber method. Deionized water (5 mL) and 25% NH_3_·H_2_O (3 mL) were added to an ethanol solution (35 mL), and stirred continuously for 0.5 h at 25 °C. Then, TEOS (5 mL) was added dropwise to the above mixture and stirring continued for 24 h. The resultant SiO_2_ nanospheres were collected by centrifugation, then washed with deionized water and ethanol and dried at 60 °C for 24 h under vacuum. (ii) The SiO_2_@mTiO_2_ core–shell nanospheres were prepared by a modified cooperative assembly-directed method. Firstly, SiO_2_ nanospheres (150 mg) were dispersed in ethanol (15 mL) by ultrasonication, then 25% NH_3_·H_2_O (0.3 mL) and HDA (150 mg) were added under stirring at room temperature. Secondly, Tip (0.3 mL) was quickly added to the above mixture and continued to stir for 1 h. The resultant core–shell SiO_2_@TiO_2_/HDA nanospheres were collected by centrifugation, then washed with deionized water and ethanol. Thirdly, SiO_2_@TiO_2_/HDA nanospheres were dispersed in a mixture of deionized water (10 mL) and ethanol (20 mL) by ultrasonication, then transferred to a 100 mL Teflon-lined autoclave and heated to 160 °C for 36 h to completely remove the HDA. Finally, the resultant SiO_2_@mTiO_2_ core–shell nanospheres were collected by centrifugation, then washed with deionized water and ethanol, dried at 60 °C for 24 h under vacuum. 

### 2.5. Preparation of SiO_2_@mTiO_2_-bpdc

To DMF (20 mL), SiO_2_@mTiO_2_ (0.10 g) and H_2_bpdc (0.30 g) were dispersed by ultrasonication, followed by refluxed at 160 °C for 6 h under stirring. The resultant SiO_2_@mTiO_2_-bpdc was collected by centrifugation, then washed with DMF and ethanol, dried at 60 °C for 24 h under vacuum. 

### 2.6. Preparation of Hybrid Material Eu(tta)_3_bpdc-SiO_2_@mTiO_2_

To ethanol (20 mL), SiO_2_@mTiO_2_-bpdc (0.10 g) and Eu(tta)_3_(H_2_O)_2_ (0.5 mmol) were dispersed by ultrasonication, followed by reflux at 80 °C for 5 h under stirring. The resultant Eu(tta)_3_bpdc-SiO_2_@mTiO_2_ was collected by centrifugation and excess unbound Eu(tta)_3_(H_2_O)_2_ was washed with ethanol until no red emission of supernatant was observed under a 254 nm UV lamp, dried at 60 °C for 24 h under vacuum.

### 2.7. Luminescent Sensing Experiment

Hybrid material Eu(tta)_3_bpdc-SiO_2_@mTiO_2_ (3 mg) was dispersed in 3 mL 0.01 M different cations ethanol solutions (K^+^, Na^+^, Li^+^, Zn^2+^, Ca^2+^, Cd^2+^, Pb^2+^, Mg^2+^, Mn^2+^, Cr^3+^ and Al^3+^) at room temperature. Then, hybrid material Eu(tta)_3_bpdc-SiO_2_@mTiO_2_ suspensions were obtained by sonicating for 30 min for luminescent measurements.

### 2.8. Characterization

Fourier transform infrared (FT-IR) spectra were measured with Bruker Tensor 27 FT-IR spectrometer (Bruker physik-AG, Karlsruhe, Germany) by using KBr pellets in the range of 400–4000 cm^−1^. Powder X-ray diffraction (PXRD) patterns were performed with Bruker D8 ADVANCE diffractometer (Bruker physik-AG) (Cu-K_α_, 1.5418 Å) at room temperature. Scanning electron microscopy (SEM) was recorded on SU8010 (Hitachi Limited, Tokyo, Japan). Transmission electron microscopy (TEM), high-resolution transmission electron microscopy (HRTEM), selected area electron diffraction (SAED) and the energy-dispersive X-ray spectroscopy (EDS) were recorded on Talos F200X (Thermo Fisher Scientific, Waltham, MA, USA). Thermogravimetric analyses (TGA) were carried out Universal V4.5A instrument (Universal Instrument, Conklin, NY, USA). Photoluminescence spectra (PL) were measured on the Hitachi F-2700 spectrophotometer (Hitachi Limited) at room temperature. The lifetimes of the samples were recorded on an Edinburgh FLSp920 fluorescence spectrometer (Edinburgh Instruments, Livingston, UK). X-ray photoelectron spectra (XPS) were measured on PHI5000 VersaProbeIII XPS (ULVAC Co. Ltd, Kanagawa, Japan) with an Al-K_α_ achromatic X-ray source. Inductively coupled plasma mass spectroscopy (ICP-MS) was performed on an Agilent 7900 ICP-MS (Agilent Technologies, Santa Clara, CA, USA).

## 3. Results and Discussion

**Scheme 1:**
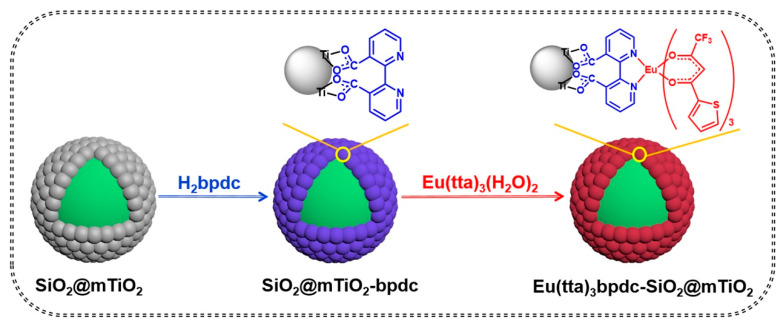
The preparation procedure and predicted structure of core–shell hybrid material Eu(tta)_3_bpdc-SiO_2_@mTiO_2_.

The preparation procedure and predicted structure of core–shell hybrid material Eu(tta)_3_bpdc-SiO_2_@mTiO_2_ are exhibited in Scheme 1. The choice of H_2_bpdc ligand is very important during the reaction process. The –COOH groups of H_2_bpdc ligand can covalently anchor onto the surface of SiO_2_@mTiO_2_ core–shell nanosphere through condensation reaction between hydroxyl groups on the surface of SiO_2_@mTiO_2_ and carboxylic groups. Meanwhile, two pyridyl N atoms of H_2_bpdc can coordinate to Eu^III^ ions in the first coordination sphere of Eu(tta)_3_(H_2_O)_2_ complex by ligand-exchange reaction and then sensitize luminescence of Eu^III^ ions with the primary ligand Htta via the “antenna effect”. Finally, complex Eu(tta)_3_bpdc can be successfully formed and covalently bonded to SiO_2_@mTiO_2_ core–shell nanospheres, then the hybrid material Eu(tta)_3_bpdc-SiO_2_@mTiO_2_ was successfully obtained.

### 3.1. FT-IR Spectra

**Figure 1:**
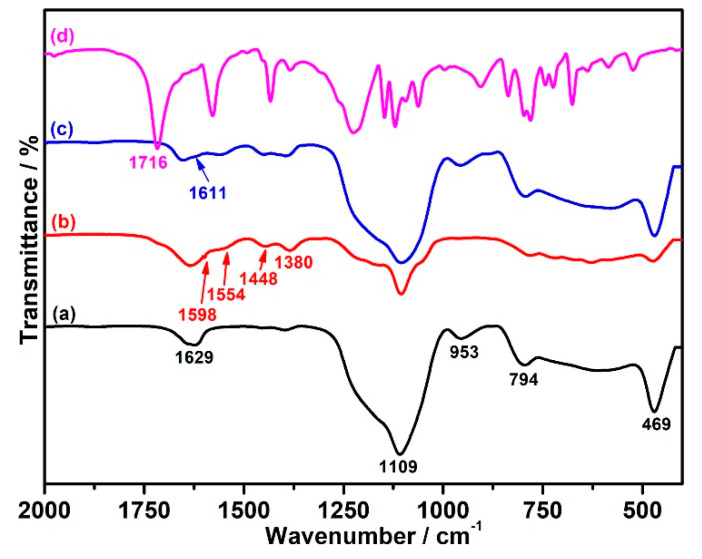
FT-IR spectra of (**a**) SiO_2_@mTiO_2_, (**b**) SiO_2_@mTiO_2_-bpdc, (**c**) Eu(tta)_3_bpdc-SiO_2_@mTiO_2_ and (**d**) H_2_bpdc ligand.

In order to confirm complex Eu(tta)_3_ successfully graft on the surface of SiO_2_@mTiO_2_ core–shell nanospheres, FT-IR spectra of H_2_bpdc ligand, SiO_2_@mTiO_2_, SiO_2_@mTiO_2_-bpdc and hybrid material Eu(tta)_3_bpdc-SiO_2_@mTiO_2_ were measured and shown in Figure 1 and Figure S1 and Table S1. In the spectra of SiO_2_@mTiO_2_ (Figure 1a), SiO_2_@mTiO_2_-bpdc (Figure 1b) and hybrid material Eu(tta)_3_bpdc-SiO_2_@mTiO_2_ (Figure 1c), the characteristic bands of Ti–O network can be observed in the range of 450~750 cm^−1^. In addition, the vibrational bands of Si–O asymmetric stretching vibration and symmetric stretching vibration appear at 1109 and 794 cm^−1^, respectively. While the peak at 953 cm^−1^ is ascribed to stretching vibration of the Si–OH surface groups. As shown in Figure 1d, H_2_bpdc ligand displays a strong band at 1716 cm^−1^ because of the stretching of carboxylic groups. The stretching of carboxylic groups is not found in the spectra of SiO_2_@mTiO_2_-bpdc (Figure 1b) and hybrid material Eu(tta)_3_bpdc-SiO_2_@mTiO_2_ (Figure 1c), indicating that the carboxylic groups of H_2_bpdc in SiO_2_@mTiO_2_-bpdc and Eu(tta)_3_bpdc-SiO_2_@mTiO_2_ were completely deprotonated. Meanwhile, the ν_asym_(COO^−^) and ν_sym_ (COO^−^) stretching vibration of carboxylate appear at 1554 and 1380 cm^−1^ and the difference (Δ = ν_asym_ − ν_sym_ = 174 cm^−1^) of carboxylate stretching frequencies can be used to identify the bonding mode, further indicating that carboxylate groups form bidentate mode with the titania in SiO_2_@mTiO_2_ nanospheres [34,35,36,37,38]. In addition, the position of stretching vibration of C=N of H_2_bpdc ligand (1611 cm^−1^) in Eu(tta)_3_bpdc-SiO_2_@mTiO_2_ has shifted compared with that in SiO_2_@mTiO_2_-bpdc (1598 cm^−1^), indicating that a possible coordination bond of Eu–N may be formed in hybrid material Eu(tta)_3_bpdc-SiO_2_@mTiO_2_. The results above confirm that complex Eu(tta)_3_ successfully graft on the surface of SiO_2_@mTiO_2_ core–shell nanospheres by a carboxylic functionalized bipyridyl ligand.

### 3.2. PXRD

**Figure 2:**
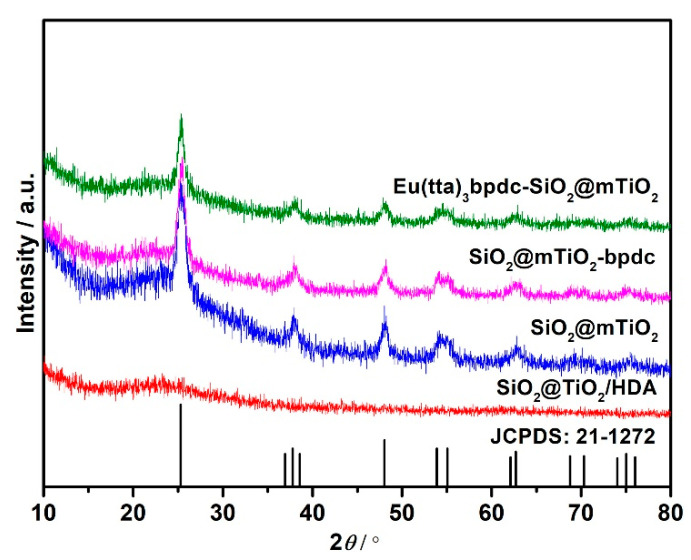
The PXRD patterns of SiO_2_@TiO_2_/HDA, SiO_2_@mTiO_2_, SiO_2_@mTiO_2_-bpdc and Eu(tta)_3_bpdc-SiO_2_@mTiO_2_.

The PXRD patterns of SiO_2_@TiO_2_/HDA, SiO_2_@mTiO_2_, SiO_2_@mTiO_2_-bpdc and Eu(tta)_3_bpdc-SiO_2_@mTiO_2_ are shown in Figure 2. The pattern of SiO_2_@TiO_2_/HDA is completely amorphous, while that of SiO_2_@mTiO_2_ exhibits an anatase phase (JCPDS card No. 21-1272), indicating that the solvothermal method can not only remove the surfactant (HDA) in material but also change the crystal phase of TiO_2_ in the shell. According to the Scherrer formula, the average crystallite size of TiO_2_ in core–shell material can be estimated to be about 22 nm. The characteristic diffraction peaks of SiO_2_@mTiO_2_ appear at 25.27, 37.84, 48.03, 54.23, 55.04, 62.64, 69.32, 71.29 and 75.63° and correspond to the (101), (004), (200), (105), (211), (204), (116), (220) and (215) diffractions of anatase phase (JCPDS card No. 21-1272), respectively. In addition, characteristic diffraction peaks of SiO_2_@mTiO_2_-bpdc and Eu(tta)_3_bpdc-SiO_2_@mTiO_2_ (Table S2) are nearly unchanged compared with those of SiO_2_@mTiO_2_, indicating that the anatase phase was maintained well after complex Eu(tta)_3_bpdc was introduced.

### 3.3. SEM Images

**Figure 3:**
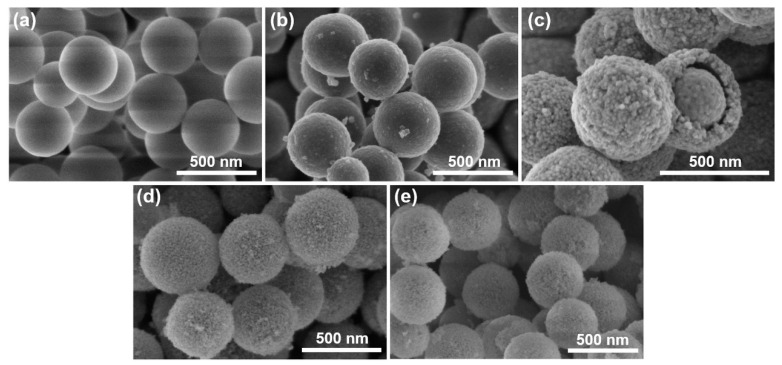
SEM images of (**a**) SiO_2_, (**b**) SiO_2_@TiO_2_/HDA, (**c**) SiO_2_@mTiO_2_, (**d**) SiO_2_@mTiO_2_-bpdc and (**e**) Eu(tta)_3_bpdc-SiO_2_@mTiO_2_.

The SEM images of SiO_2_, SiO_2_@TiO_2_/HDA, SiO_2_@mTiO_2_, SiO_2_@mTiO_2_-bpdc and Eu(tta)_3_bpdc-SiO_2_@mTiO_2_ are displayed in Figure 3. It can be observed that SiO_2_, SiO_2_@TiO_2_/HDA, SiO_2_@mTiO_2_, SiO_2_@mTiO_2_-bpdc and Eu(tta)_3_bpdc-SiO_2_@mTiO_2_ show monodisperse spherical structures. The average diameter of SiO_2_ is about 350 nm, and that of SiO_2_@TiO_2_/HDA, SiO_2_@mTiO_2_, SiO_2_@mTiO_2_-bpdc and Eu(tta)_3_bpdc-SiO_2_@mTiO_2_ is about 430 nm, the thickness of the shell in the core–shell structure is about 40 nm. The surfactant (HDA) in SiO_2_@TiO_2_/HDA materials were removed by solvothermal method, then SiO_2_@mTiO_2_ core–shell nanospheres were obtained. Meanwhile, it is found that the shell of SiO_2_@mTiO_2_ becomes rough and consists of many small nanoparticles. The surface morphologies and sizes of SiO_2_@mTiO_2_-bpdc and Eu(tta)_3_bpdc-SiO_2_@mTiO_2_ are nearly unchanged in comparison with those of SiO_2_@mTiO_2_, suggesting that the load of the europium complex does not influence the morphology or size of SiO_2_@mTiO_2_.

### 3.4. TEM Images

**Figure 4:**
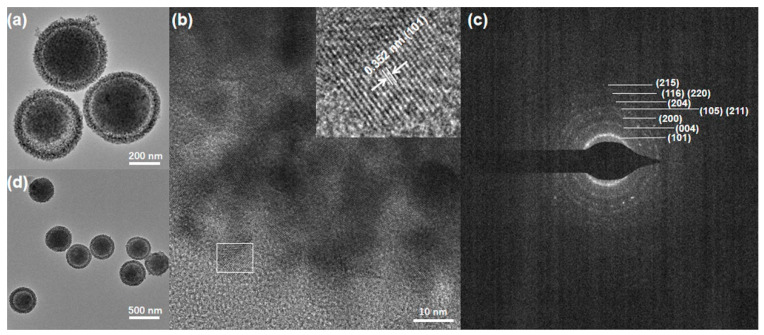
(**a)** TEM image of SiO_2_@mTiO_2_, (**b**) HRTEM image of the anatase crystals of shell in SiO_2_@mTiO_2_, (**c**) the corresponding SAED pattern of SiO_2_@mTiO_2_ and (**d**) TEM image of Eu(tta)_3_bpdc-SiO_2_@mTiO_2_.

**Figure 5:**
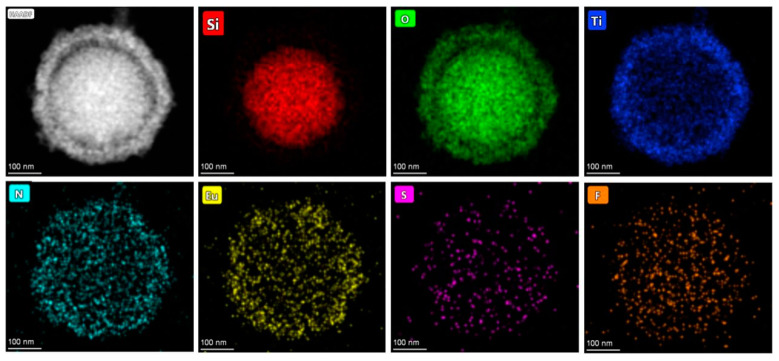
EDS mapping for each element in Eu(tta)_3_bpdc-SiO_2_@mTiO_2_.

The TEM, HRTEM and SAED images of SiO_2_@mTiO_2_ and the TEM image of hybrid material Eu(tta)_3_bpdc-SiO_2_@mTiO_2_ are shown in Figure 4. SiO_2_@mTiO_2_ (Figure 4a) and Eu(tta)_3_bpdc-SiO_2_@mTiO_2_ (Figure 4d) exhibit monodisperse spherical core–shell structures with an average diameter of about 430 nm. The shell of SiO_2_@mTiO_2_ and Eu(tta)_3_bpdc-SiO_2_@mTiO_2_ is comprised of many small-ordered nanoparticles (Figure S2). The HRTEM can be used to identify the nature of nanocrystalline titania in SiO_2_@mTiO_2_ (Figure 4b). The fringe spacing is about 3.52 Å, corresponding to the (101) plane of anatase titania, further confirming that the shell of the nanosphere consists of single anatase nano-crystallites. As shown in Figure 4c, the SAED pattern clearly shows several continuous rings, corresponding to the (101), (004), (200), (105/211), (204), (116/220) and (215) diffractions of the anatase phase. In addition, the morphology and size of the hybrid material Eu(tta)_3_bpdc-SiO_2_@mTiO_2_ are nearly unchanged in comparison with those of SiO_2_@mTiO_2_, indicating that the introduction of europium complex does not influence the morphology and size of the SiO_2_@mTiO_2_ matrices. The composition and distribution of the elements in SiO_2_@mTiO_2_ and Eu(tta)_3_bpdc-SiO_2_@mTiO_2_ were analyzed by EDS mapping (Figure S3 and Figure 5). As displayed in Figure S3, Si and Ti elements are uniformly distributed in the core and shell, suggesting that SiO_2_@mTiO_2_ is a core–shell structure of silica coated by titania. Furthermore, for hybrid material Eu(tta)_3_bpdc-SiO_2_@mTiO_2_ (Figure 5), a uniform dispersity of N, Eu, S and F elements indicates the existence of complex Eu(tta)_3_bpdc in core–shell nanosphere, which further suggests the hybrid material was successfully obtained.

### 3.5. Luminescent Properties

**Figure 6:**
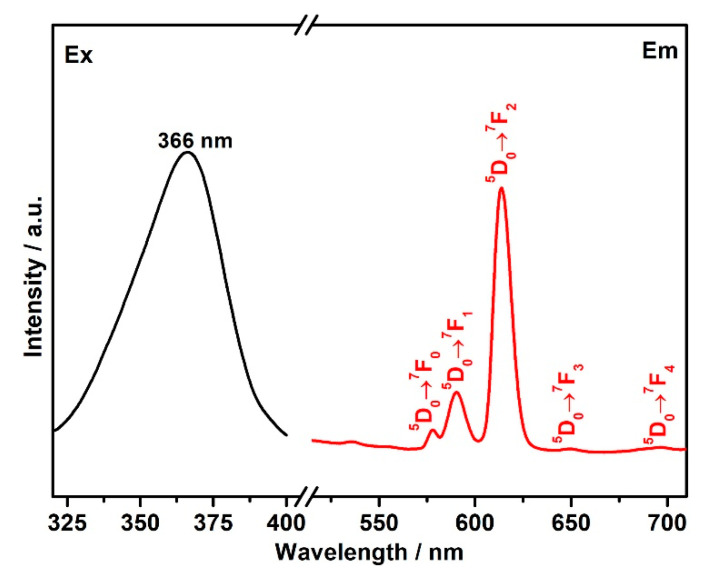
Excitation and emission spectra of hybrid material Eu(tta)_3_bpdc-SiO_2_@mTiO_2_ in solid state at room temperature.

The luminescent behavior of hybrid material Eu(tta)_3_bpdc-SiO_2_@mTiO_2_ in solid state and ethanol solution was investigated at room temperature. The excitation spectra of hybrid material Eu(tta)_3_bpdc-SiO_2_@mTiO_2_ are obtained by monitoring the most intense ^5^D_0_ → ^7^F_2_ emission. As presented in Figure 6, the excitation spectrum shows a broad band in the range of 300~400 nm, which was attributed to the π → π* electron transition of H_2_bpdc ligand. Upon the maximum excitation (λ_ex_ = 366 nm), the emission spectrum of hybrid material Eu(tta)_3_bpdc-SiO_2_@mTiO_2_ in a solid state presents characteristic ^5^D_0_ → ^7^F_J_ (J = 0, 1, 2, 3, 4) transitions of Eu^III^ ions in the range of 550~710 nm, indicating that the effective energy transfer is occurred from the ligands (H_2_bpdc and Htta) to the central Eu^III^ ion, further implying that core–shell SiO_2_@mTiO_2_ nanospheres can act as an efficient host to sensitize the luminescence of Eu^III^ ion. The ^5^D_0_ → ^7^F_2_ emission intensity of Eu^III^ is strongly dependent on the environment because of its electric-dipole character, while the intensity of the ^5^D_0_ → ^7^F_1_ transition is independent of the environment due to its magnetic dipole character. The intensity ratios *I*(^5^D_0_ → ^7^F_2_)/*I*(^5^D_0_ → ^7^F_1_) will provide valuable information about the surrounding environment changes around Eu^III^ ion. The ratio in complex Eu(tta)_3_(H_2_O)_2_ is about 7.7, while that in hybrid material Eu(tta)_3_bpdc-SiO_2_@mTiO_2_ is about 3.9, the decrease in the ratio demonstrates that the symmetry of the coordination environment around the Eu^III^ ions has changed during the formation of hybrid material Eu(tta)_3_bpdc-SiO_2_@mTiO_2_, which further indicates that the tripyridyl group replaces the coordination water molecules in the first coordination sphere of Eu^III^ ions in a complex by ligand-exchange reaction, and the coordination bonds of Eu–N are formed. The ^5^D_0_ luminescent decay curve was recorded at 613 nm under the maximum excitation (λ_ex_ = 366 nm) and is exhibited in Figure S4. The curve can be fitted by biexponential functions, the average lifetime (*τ*) can be calculated using the following equation:

(1) $$\tau =\frac{\sum A_{i}\tau_{i}^{2}}{\sum A_{i}\tau_{i}},$$

where *τ_i_* is the component decay times and *A_i_* is the preexponential factors related to the statistical weights of each exponential. For hybrid material Eu(tta)_3_bpdc-SiO_2_@mTiO_2_, the obtained ^5^D_0_ lifetime value was 0.52 ms.

As shown in Figure S5, hybrid material Eu(tta)_3_bpdc-SiO_2_@mTiO_2_ in an ethanol solution also shows five characteristic ^5^D_0_ → ^7^F_J_ (J = 0, 1, 2, 3, 4) emission peaks of Eu^III^ ions and shows luminous red color emission. At the same time, Eu(tta)_3_bpdc-SiO_2_@mTiO_2_ in an ethanol solution possesses excellent luminescent stability at room temperature. In addition, hybrid material Eu(tta)_3_bpdc-SiO_2_@mTiO_2_ also has a nanoscale nature, so it can act as a suitable luminescent sensor in environmental and biological systems.

### 3.6. Sensing of Al^3+^ Cations

**Figure 7:**
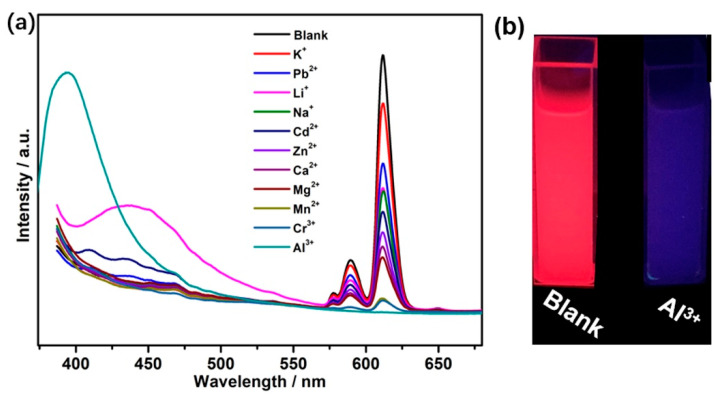
(**a**) Photoluminescence spectra (λ_ex_ = 367 nm) of hybrid material Eu(tta)_3_bpdc-SiO_2_@mTiO_2_ after dispersed in ethanol solutions of different metal ions and (**b**) pictures of hybrid material Eu(tta)_3_bpdc-SiO_2_@mTiO_2_ under the 365 nm UV lamp after dispersed in ethanol solutions without and with Al^3+^ ions.

In order to investigate the potential application of core–shell hybrid material Eu(tta)_3_bpdc-SiO_2_@mTiO_2_ for the sensing of metal ions, Eu(tta)_3_bpdc-SiO_2_@mTiO_2_ powder samples were dispersed in ethanol solutions of different metal ions (KCl, NaCl, LiCl, ZnCl_2_, CaCl_2_, CdCl_2_, PbCl_2_, MgCl_2_, MnCl_2_, CrCl_3_ and AlCl_3_) for luminescent measurements. The luminescent emission of samples was recorded and presented in Figure 7a. Most of these cations influence the emission intensities of hybrid material Eu(tta)_3_bpdc-SiO_2_@mTiO_2_, especially for Mn^2+^ and Cr^3+^ ions show a significant quenching effect on the luminescent intensity. These phenomena are caused by the different electron configuration of metal ions: K^+^, Na^+^, Li^+^, Zn^2+^, Ca^2+^, Cd^2+^, Pb^2+^ and Mg^2+^ with a closed-shell electron configuration have much weaker effects on luminescent intensity, whereas other ions (such as Mn^2+^ and Cr^3+^) have different electron configurations and produce varying degrees of quenching of luminescent intensity [39]. However, only Al^3+^ ions have a significant impact on the emission spectrum of hybrid material Eu(tta)_3_bpdc-SiO_2_@mTiO_2_, characteristic emission of Eu^III^ ions disappears, a broad band of H_2_bpdc ligand appears at 396 nm, and the luminescent color has clearly changed from red to blue under UV-light irradiation (Figure 7b). The reason for the selective recognition of hybrid material Eu(tta)_3_bpdc-SiO_2_@mTiO_2_ toward Al^3+^ ions may be that there existed stronger coordination interaction between bpdc^2^^−^ ligand and Al^3+^ ions than bpdc^2−^ ligand and Eu^III^ ions [40].

**Figure 8:**
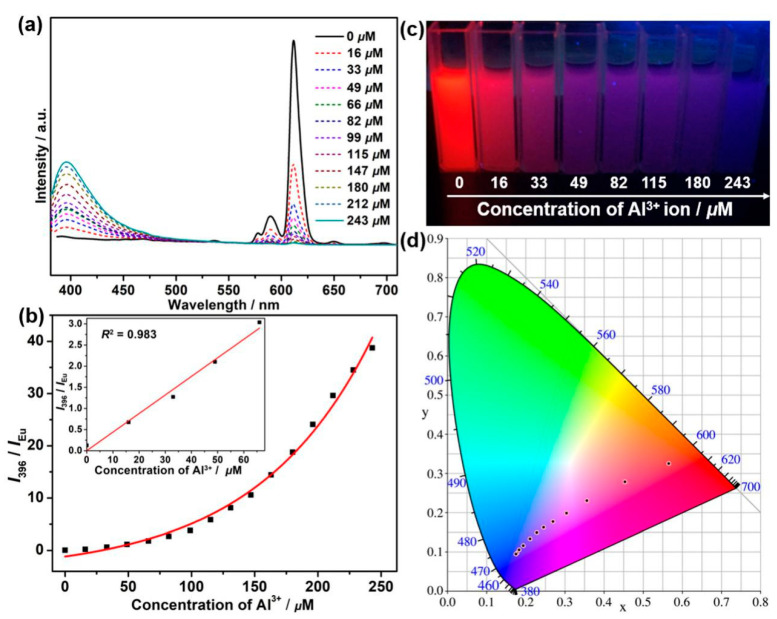
(**a**) Photoluminescence spectra; (**b**) the plot of emission intensity of 396 nm/613 nm vs. concentration of Al^3+^ ions (Inset: the linear correlation for the plot of emission intensity of 396 nm/613 nm vs. concentration of Al^3+^ ions); (**c**) the pictures of hybrid material Eu(tta)_3_bpdc-SiO_2_@mTiO_2_ in different concentrations of Al^3+^ ions under the 365 nm UV lamp; (**d**) the corresponding CIE chromaticity diagram of hybrid material Eu(tta)_3_bpdc-SiO_2_@mTiO_2_ in an ethanol solution with different concentrations of Al^3+^ ions (λ_ex_ = 367 nm).

A luminescent titration experiment was carried out to evaluate the luminescent response of hybrid material Eu(tta)_3_bpdc-SiO_2_@mTiO_2_ to Al^3+^ ions by the addition of Al^3+^ ions to an Eu(tta)_3_bpdc-SiO_2_@mTiO_2_ ethanol solution. As shown in Figure 8a, with the increase in Al^3+^ ion concentration, the luminescent intensity of Eu^III^ ions at 613 nm gradually weakens; meanwhile, a broad band of H_2_bpdc ligand at 396 nm appears and gradually enhances, leading to emission color change from red to pink, purplish pink, purplish blue, and finally blue under the 365 nm UV lamp (Figure 8c). The distinguishable colors at different concentrations of Al^3+^ ions are attributed to the changeable rate of the luminescent relative intensity of H_2_bpdc ligand-centered emissions and the characteristic emission of Eu^III^ ions. Hence, the naked-eye recognition of Al^3+^ ions is feasible according to the color change of hybrid material Eu(tta)_3_bpdc-SiO_2_@mTiO_2_ ethanol solution. As a result, by changing the concentration of Al^3+^ ions in hybrid material Eu(tta)_3_bpdc-SiO_2_@mTiO_2_ ethanol solution, the relative emission intensities of two constituent colors (red and blue) can be precisely manipulated, resulting in a tunable multicolor output. The emission color CIE coordinates of hybrid material Eu(tta)_3_bpdc-SiO_2_@mTiO_2_ ethanol solution with various concentrations of Al^3+^ ions excited at 367 nm in the CIE 1931 chromaticity diagram (Figure 8d) and listed in Table S3. When Al^3+^ ion concentration increases to 243 μM, the red emission from Eu^III^ ions almost completely quenches. Noticeably, a linear relationship is observed at low concentrations between the emission intensity ratio of 396 nm/613 nm and the concentration of Al^3+^ ions, followed the simple linear equation of y = 0.0439x + 0.0011 (*R*^2^ = 0.983) (Figure 8b inset). The limit of detection (LOD) for Al^3+^ ions was determined as 1.8 × 10^−4^ M according to the equation LOD = 3σ/k. The result indicates that hybrid material Eu(tta)_3_bpdc-SiO_2_@mTiO_2_ can be acted as a highly sensitive and selective probe for the quantitative luminescent detection of Al^3+^ ions in an ethanol solution.

To assess the selectivity of hybrid material Eu(tta)_3_bpdc-SiO_2_@mTiO_2_, the competitive tests of hybrid material Eu(tta)_3_bpdc-SiO_2_@mTiO_2_ with Al^3+^ and different metal ions were carried out in an ethanol solution. As shown in Figure S6, after adding other ions in Al^3+^ ethanol solution, the luminescent intensity of hybrid material Eu(tta)_3_bpdc-SiO_2_@mTiO_2_ at 396 nm increases compared with that in Al^3+^ ethanol solution. The result suggests that the detection of Al^3+^ ions is not affected by the other ions.

**Figure 9:**
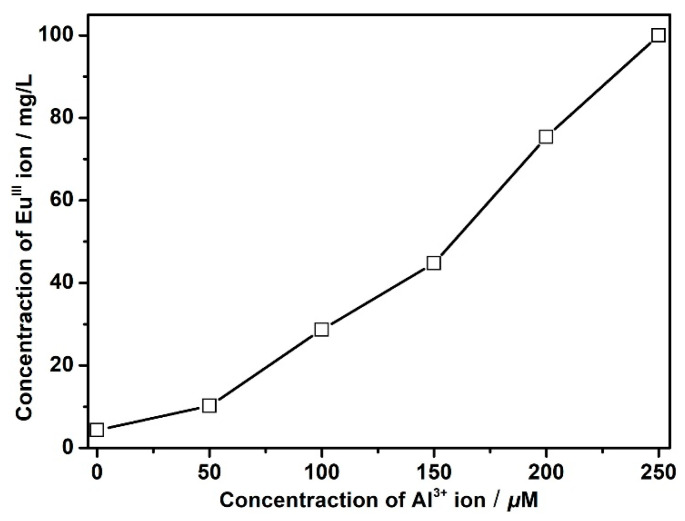
The concentrations of Eu^III^ ions in the filtrate of Eu(tta)_3_bpdc-SiO_2_@mTiO_2_ upon the addition of different contents of Al^3+^ ions.

**Figure 10:**
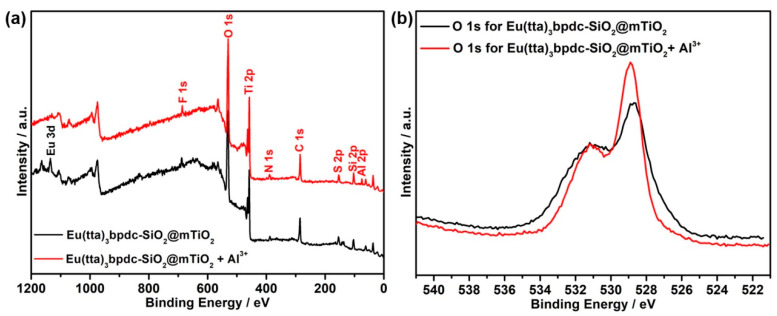
(**a**) XPS survey spectra for hybrid materials Eu(tta)_3_bpdc-SiO_2_@mTiO_2_ and Eu(tta)_3_bpdc-SiO_2_@mTiO_2_ + Al^3+^, (**b**) O 1s spectra for hybrid materials Eu(tta)_3_bpdc-SiO_2_@mTiO_2_ and Eu(tta)_3_bpdc-SiO_2_@mTiO_2_ + Al^3+^.

According to the reported literature [16,30], the possible luminescent sensing mechanism for the detection of Al^3+^ ions originates from two aspects: (i) the cation exchange of central lanthanide ions in lanthanide hybrid materials with targeted cations, (ii) the interaction between organic ligands and Al^3+^ ions. Therefore, in order to verify this hypothesis, ICP-MS, XPS, PXRD, TEM and EDS mapping of hybrid material Eu(tta)_3_bpdc-SiO_2_@mTiO_2_ after immersed in an Al^3+^ ion ethanol solution (namely, Eu(tta)_3_bpdc-SiO_2_@mTiO_2_ + Al^3+^) were carried out. As exhibited in Figure 9, as the content of added Al^3+^ ions in the filtrate increases, the concentration of Eu^III^ ions in the filtrate gradually increases. As a result, Al^3+^ ions may gradually replace Eu^III^ ions in hybrid material Eu(tta)_3_bpdc-SiO_2_@mTiO_2_, which corresponds to luminescence responses of hybrid material Eu(tta)_3_bpdc-SiO_2_@mTiO_2_ toward Al^3+^ ions in ethanol solutions with varying concentrations of Al^3+^ ions, indicating that the luminescent emission changes are attributed to the cation exchange. As shown in Figure 10 and Figure S7 and Table S4, it is clearly observed that Eu 3p peak (~1138.4 eV) disappears and Al 2p peak (~74.8 eV) appears in Eu(tta)_3_bpdc-SiO_2_@mTiO_2_ + Al^3+^ when compared with those peaks in hybrid material Eu(tta)_3_bpdc-SiO_2_@mTiO_2_, indicating that Eu^III^ ions in hybrid material Eu(tta)_3_bpdc-SiO_2_@mTiO_2_ were gradually replaced by Al^3+^ ions in an ethanol solution, further suggesting that there existed a cation exchange when hybrid material Eu(tta)_3_bpdc-SiO_2_@mTiO_2_ was immersed in an ethanol solution of Al^3+^ ions. In addition, the O 1s peak [41] in the spectrum of Eu(tta)_3_bpdc-SiO_2_@mTiO_2_ + Al^3+^ almost have no shift compared with that in the spectrum of hybrid material Eu(tta)_3_bpdc-SiO_2_@mTiO_2_ (Figure 10b), to some content, which excludes the possibility of interaction between oxygen atoms in coordinated carboxylate group of bpdc^2−^ ligand and Al^3+^ ions. As displayed in Figure S8 and Table S2, characteristic diffraction peaks of Eu(tta)_3_bpdc-SiO_2_@mTiO_2_ + Al^3+^ materials are nearly unchanged compared with those of hybrid material Eu(tta)_3_bpdc-SiO_2_@mTiO_2_, indicating that the anatase phase was maintained well after hybrid material Eu(tta)_3_bpdc-SiO_2_@mTiO_2_ was immersed in an ethanol solution of Al^3+^ ions. Additionally, it can be observed that Eu(tta)_3_bpdc-SiO_2_@mTiO_2_ + Al^3+^ shows the core–shell structure (Figure S9) and the size of the core–shell nanosphere of Eu(tta)_3_bpdc-SiO_2_@mTiO_2_ + Al^3+^ almost have no change compared with that of Eu(tta)_3_bpdc-SiO_2_@mTiO_2_. This suggests that the subsequent addition of Al^3+^ does not nearly change the morphology or size of the hybrid material Eu(tta)_3_bpdc-SiO_2_@mTiO_2_. As shown in Table S5, the Al element could be seen besides Si, O, Ti, N, F, S, and mass ratio of Eu element decreases and approaches zero compared with the mass ratio of the Eu element in hybrid material Eu(tta)_3_bpdc-SiO_2_@mTiO_2_, which suggests that Eu^III^ ions in Eu(tta)_3_bpdc-SiO_2_@mTiO_2_ were replaced by Al^3+^ ions, and then excludes interaction between the ligands of Eu(tta)_3_bpdc-SiO_2_@mTiO_2_ and Al^3+^ ions. Furthermore, Si, O, Ti, N, F, S and Al elements are homogeneously distributed in the hybrid material Eu(tta)_3_bpdc-SiO_2_@mTiO_2_ + Al^3+^ (Figure S10). Based on the above results, the possible luminescence sensing mechanism for the detection of Al^3+^ ions originates from the cation exchange between Eu^III^ ions in hybrid material Eu(tta)_3_bpdc-SiO_2_@mTiO_2_ and Al^3+^ ions.

## 4. Conclusions

A europium(III) hybrid material based on a SiO_2_@mTiO_2_ core–shell nanosphere by covalent grafting complex Eu(tta)_3_bpdc was successfully designed and synthesized. H_2_bpdc ligand can act as a bifunctional linker to graft Eu(tta)_3_ to the surface of the core–shell SiO_2_@mTiO_2_ nanosphere. The results indicate that core–shell structure and anatase crystallites are retained after grafting to the europium complex. Hybrid material Eu(tta)_3_bpdc-SiO_2_@mTiO_2_ displays uniform nanosphere structure, bright red color emission and long lifetime, which can sever as a multicolor emission material to modulate by using Al^3+^ ions via an ion exchange approach under a single-wavelength excitation.

## Supplementary Materials

The following are available online at www.mdpi.com/article/10.3390/nano11112886/s1, Figure S1: FT-IR spectra of SiO_2_@mTiO_2_ (a), SiO_2_@mTiO_2_-bpdc (b), Eu(tta)_3_bpdc-SiO_2_@mTiO_2_ (c) and H_2_bpdc ligand (d); Figure S2: TEM image of SiO_2_@mTiO_2_; Figure S3: EDS mapping for each element in SiO_2_@mTiO_2_; Figure S4: Luminescent lifetime of hybrid material Eu(tta)_3_bpdc-SiO_2_@mTiO_2_ in state solid; Figure S5: Excitation and emission spectra of hybrid material Eu(tta)_3_bpdc-SiO_2_@mTiO_2_ in ethanol solution at room temperature; Figure S6: Luminescent intensities of hybrid material Eu(tta)_3_bpdc-SiO_2_@mTiO_2_ at 396 nm in presence of other metal ions and Al^3+^ ion in ethanol solution; Figure S7: C 1s spectra for hybrid materials Eu(tta)_3_bpdc-SiO_2_@mTiO_2_ and Eu(tta)_3_bpdc-SiO_2_@mTiO_2_ + Al^3+^; Figure S8: The PXRD patterns of hybrid materials Eu(tta)_3_bpdc-SiO_2_@mTiO_2_ and Eu(tta)_3_bpdc-SiO_2_@mTiO_2_ + Al^3+^; Figure S9: TEM image of Eu(tta)_3_bpdc-SiO_2_@mTiO_2_ + Al^3+^; Figure S10: EDS mapping for each element in Eu(tta)_3_bpdc-SiO_2_@mTiO_2_ + Al^3+^. Table S1: The FT-IR characteristic bands of SiO_2_@mTiO_2_, SiO_2_@mTiO_2_-bpdc, Eu(tta)_3_bpdc-SiO_2_@mTiO_2_ and H_2_bpdc ligand; Table S2: The characteristic diffraction peaks for PXRD in SiO_2_@mTiO_2_, SiO_2_@mTiO_2_-bpdc, Eu(tta)_3_bpdc-SiO_2_@mTiO_2_ and Eu(tta)_3_bpdc-SiO_2_@mTiO_2_ + Al^3+^; Table S3: CIE coordinates and emission colors of hybrid material Eu(tta)_3_bpdc-SiO_2_@mTiO_2_ in different concentrations of Al^3+^ ion; Table S4: Binding energies for XPS in hybrid materials Eu(tta)_3_bpdc-SiO_2_@mTiO_2_ and Eu(tta)_3_bpdc-SiO_2_@mTiO_2_ + Al^3+^; Table S5: The mass ratio of each element in Eu(tta)_3_bpdc-SiO_2_@mTiO_2_ and Eu(tta)_3_bpdc-SiO_2_@mTiO_2_ + Al^3+^. Reference [42] is cited in the supplementary materials.

## References

[1] Ning Y., Zhu M., Zhang J.-L. (2019). Near-infrared (NIR) lanthanide molecular probes for bioimaging and biosensing. Coord. Chem. Rev..

[2] Chen H., Cao J., Zhou P., Li X., Xie Y., Liu W., Tang Y. (2018). Multiplex recognition and logic devices for molecular robot prototype based on an europium(iii)-cyclen system. Biosens. Bioelectron..

[3] Dou Z.-S., Yu J.-C., Cui Y.-J., Yang Y., Wang Z.-Y., Yang D.-R., Qian G.-D. (2014). Luminescent Metal-Organic Framework Films As Highly Sensitive and Fast-Response Oxygen Sensors. J. Am. Chem. Soc..

[4] Chen L., Liu D., Peng J., Du Q., He H. (2020). Ratiometric fluorescence sensing of metal-organic frameworks: Tactics and perspectives. Coord. Chem. Rev..

[5] Cui Y., Chen B., Qian G. (2014). Lanthanide metal-organic frameworks for luminescent sensing and light-emitting applications. Coord. Chem. Rev..

[6] SeethaLekshmi S., Ramya A.R., Reddy M.L.P., Varughesea S. (2017). Lanthanide complex-derived white-light emitting solids: A survey on design strategies. J. Photoch. Photobiol. C.

[7] Bünzli J.-C.G., Piguet C. (2005). Taking advantage of luminescent lanthanide ions. Chem. Soc. Rev..

[8] Ou Y., Zhou W., Zhu Z., Ma F., Zhou R., Su F., Zheng L., Ma L., Liang H. (2020). Host Differential Sensitization toward Color/Lifetime-Tuned Lanthanide Coordination Polymers for Optical Multiplexin. Angew. Chem. Int. Ed..

[9] Zhang W., Zhang Y.-M., Xie F., Jin X., Li J., Yang G., Gu C., Wang Y., Zhang S.X.-A. (2020). A Single-Pixel RGB Device in a Colorful Alphanumeric Electrofluorochromic Display. Adv. Mater..

[10] Gao W., Wang R., Han Q., Dong J., Yan L., Zheng H. (2015). Tuning Red Upconversion Emission in Single LiYF_4_:Yb^3+^/Ho^3+^ Microparticle. J. Phys. Chem. C.

[11] Jose B.A.S., Matsushita S., Akagi K. (2012). Lyotropic Chiral Nematic Liquid Crystalline Aliphatic Conjugated Polymers Based on Disubstituted Polyacetylene Derivatives That Exhibit High Dissymmetry Factors in Circularly Polarized Luminescence. J. Am. Chem. Soc..

[12] Li G.G., Hou Z.Y., Peng C., Wang W.X., Cheng Z.Y., Li C.X., Lian H.Z., Lin J. (2010). Electrospinning Derived One-Dimensional LaOCl: Ln^3+^ (Ln = Eu/Sm, Tb, Tm) Nanofibers, Nanotubes and Microbelts with Multicolor-Tunable Emission Properties. Adv. Funct. Mater..

[13] He G.J., Guo D., He C., Zhang X.L., Zhao X.W., Duan C.Y. (2009). A color-tunable europium complex emitting three primary colors and white light. Angew. Chem. Int. Ed..

[14] Cui Y.J., Xu H., Yue Y.F., Guo Z.Y., Yu J.C., Chen Z.X., Gao J.K., Yang Y., Qian G.D., Chen B.L. (2012). A luminescent mixed-lanthanide metal-organic framework thermometer. J. Am. Chem. Soc..

[15] Dang S., Zhang J.H., Sun Z.M. (2012). Tunable emission based on lanthanide(III) metal-organic frameworks: An alternative approach to white light. J. Mater. Chem..

[16] Zhang Z., Li H., Li Y., Yu X. (2019). Full-color emission of a Eu^3+^-based mesoporous hybrid material modulated by Zn^2+^ ions: Emission color changes for Zn^2+^ sensing via an ion exchange approach. Dalton Trans..

[17] Li Y., Yu X., Yu T. (2017). Eu^3+^ based mesoporous hybrid material with tunable multicolor emission modulated by fluoride ion: Application for selective sensing toward fluoride ion. J. Mater. Chem. C.

[18] Perl D.P., Brody A.R. (1980). Alzheimer’s disease: X-ray spectrometric evidence of aluminum accumulation in neurofibrillary tangle-bearing neurons. Science.

[19] Barcelo J., Poschenrieder C. (2002). Fast root growth responses, root exudates, and internal detoxification as clues to the mechanisms of aluminum toxicity and resistance. Exp. Bot..

[20] Valeur B., Leray I. (2000). Design principles of fluorescent molecular sensors for cation recognition. Coord. Chem. Rev..

[21] Krejpcio Z., Wojciak R.W.P.J. (2002). The Influence of Al^3+^ Ions on Pepsin and Trypsin Activity in Vitro. Environ. Stud..

[22] Goswami S., Paul S., Manna A. (2013). Selective “naked eye” detection of Al(iii) and PPi in aqueous media on a rhodamine-isatin hybrid moiety. RSC Adv..

[23] Kashyap K.S., Kumar A., Hira S.K., Dey S. (2019). Recognition of Al^3+^ through the off-on mechanism as a proficient driving force for the hydrolysis of BODIPY conjugated Schiff base and its application in bio-imaging. Inorg. Chim. Acta.

[24] Kim H., Manivannan R., Son Y.-A. (2020). A Chromone Based Fluorescent Probe for the Effective Detection of Aluminium Ion. J. Nanosci. Nanotechnol..

[25] Park J., Angupillai S., Son Y.-A. (2015). A Highly Sensitive Fluorescent Probe for Selective Detection of Al^3+^ Cation by Switching the Solvent from Aprotic to Protic Environment. Mol. Cryst. Liq. Cryst..

[26] Thangaraja S.E., Antonya E.J., Selvanb G.T., Selvakumarb P.M., Enocha I.V.M.V. (2019). A New Fluorenone-Based Turn-on Fluorescent Al^3+^ Ion Sensor. J. Anal. Chem..

[27] Ma D., Chen C., Chen M., Zhu S., Wu Y., Li Z., Li Y., Zhou L. (2019). A hydrostable Cadmium-Organic Framework for Highly Selective and Sensitive Luminescence Sensing of Al^3+^ Ion. J. Inorg. Organomet. Polym. Mater..

[28] Xu W., Zhou Y., Huang D., Su M., Wang K., Hong M. (2014). A highly sensitive and selective fluorescent sensor for detection of Al^3+^ using a Europium(III) quinolinecarboxylate. Inorg Chem..

[29] Song H., Liu G., Fan C., Pu S. (2021). A novel fluorescent sensor for Al^3+^ and Zn^2+^ based on a new europium complex with a 1,10-phenanthroline ligand. J. Rare Earth.

[30] Li H., Li Y., Zhang Z., Pang X., Yu X. (2019). Highly selective luminescent sensing of Cu^2+^ in aqueous solution based on a Eu(III)-centered periodic mesoporous organosilicas hybrid. Mater. Design.

[31] Liu Y., Sun L., Liu J., Peng Y.-X., Ge X., Shi L., Huang W. (2015). Multicolor (Vis-NIR) mesoporous silica nanospheres linked with lanthanide complexes using 2-(5-bromothiophen)imidazo[4,5-f][1,10]phenanthroline for in vitro bioimaging. Dalton Trans..

[32] Guan B.Y., Yu L., Li J., Lou X.W. (2016). A universal cooperative assembly-directed method for coating of mesoporous TiO_2_ nanoshells with enhanced lithium storage properties. Sci. Adv..

[33] Xia Q., Huang Y., Xiao J., Wang L., Lin Z., Li W., Liu H., Gu Q., Liu H.K., Chou S.-L. (2019). Phosphorus-Modulation-Triggered Surface Disorder in Titanium Dioxide Nanocrystals Enables Exceptional Sodium-Storage Performance. Angew. Chem. Int. Ed..

[34] Liu P., Li H.R., Wang Y.G., Liu B.Y., Zhang W.J., Wang Y.J., Yan W.D., Zhang H.J., Schubert U. (2008). Europium complexes immobilization on titania via chemical modification of titanium alkoxide. J. Mater. Chem..

[35] Nazeeruddin M.K., Humphry-Baker R., Liska P., Grätzel M. (2003). Investigation of Sensitizer Adsorption and the Influence of Protons on Current and Voltage of a Dye-Sensitized Nanocrystalline TiO_2_ Solar Cell. J. Phys. Chem. B.

[36] Nakamoto K. (1978). Infrared and Raman Spectra of Inorganic and Coordination Compounds.

[37] Bai C., Wei F.-H., Hu H.-M., Yan L., Wang X., Xue G.-L. (2020). New highly luminescent europium (III) complex covalently bonded with titania-based host via using a terpyridine carboxylate derivative linker for fluorescence sensing. J. Lumin..

[38] Wei F., Bai C., Hu H.-M., He S., Wang X., Xue G. (2021). Novel luminescent europium-centered hybrid material covalently grafted with organically modified titania via 2-substituted imidazophenanthroline for fluorescence sensing. J. Rare Earth.

[39] Aleem A.R., Liu J., Wang J., Wang J., Zhao Y., Wang Y., Wang Y., Wang W., Rehman F., Kipper M.J. (2020). Selective Sensing of Cu^2+^ and Fe^3+^ Ions with Vis-Excitation using Fluorescent Eu^3+^-Induced Aggregates of Polysaccharides (EIAP) in Mammalian Cells and Aqueous Systems. J. Hazard. Mater..

[40] Yu X.D., Wang Z.Y., Li Y.J., Geng L.J., Ren J.J., Feng G.L. (2017). Fluorescent and Electrochemical Supramolecular Coordination Polymer Hydrogels Formed from Ion-Tuned Self-Assembly of Small Bis-Terpyridine Monomer. Inorg. Chem..

[41] Orudzhev F., Ramazanov S., Sobola D., Isaev A., Wang C., Magomedova A., Kadiev M., Kaviyarasu K. (2020). Atomic Layer Deposition of Mixed-Layered Aurivillius Phase on TiO_2_ Nanotubes: Synthesis, Characterization and Photoelectrocatalytic Properties. Nanomaterials.

[42] Sun L., Wang Z., Zhang J.Z., Feng J., Liu J., Zhao Y., Shi L. (2014). Visible and near-infrared luminescent mesoporous titania mi-crospheres functionalized with lanthanide complexes: Microstructure and luminescence with visible excitation. RSC Adv..

