# Supplementary material for: Europium(III) Complex-Functionalized SiO2@mTiO2 Nanospheres for Al3+-Modulated Multicolor Emission
Source: Nanomaterials (Basel). 2021 Oct 28;11(11):2886. doi: 10.3390/nano11112886 (PMC8618485; doi:10.3390/nano11112886)
Supplement: Supplementary file 1 [file nanomaterials-11-02886-s001.zip › nanomaterials-1435090-supplementary.pdf]

nanomaterials

Supplementary Materials

Europium(III) Complex-Functionalized SiO_2@mTiO_2 Nanospheres for Al^{3+}-Modulated Multicolor Emission

Chao Bai [1,2], Shi He [2], Huai-Ming Hu [2,*], Hui Zeng [2], Feng Zou [2] and Ji-Jiang Wang [1,*]

[1] Key Laboratory of New Energy & New Functional Materials, Shaanxi Key Laboratory of Chemical Reaction Engineering, College of Chemistry and Chemical Engineering, Yan'an University, Yan'an 716000, China; baichao_chem@163.com

[2] Key Laboratory of Synthetic and Natural Functional Molecule of the Ministry of Education, College of Chemistry and Materials Science, Northwest University, Xi'an 710127, China; hans5113@163.com (S.H.); 17863522138@163.com (H.Z.); 201931739@stumail.nwu.edu. (F.Z.)

* Correspondence: Chemhu1@nwu.edu.cn (H.-M.H.); yadxwjj@126.com (J.-J.W.)

Table of Contents

1. **Figure S1.** FT-IR spectra of SiO_2@mTiO_2 (a), SiO_2@mTiO_2-bpdc (b), Eu(tta)$_3$bpdc-SiO_2@mTiO_2 (c) and H_2bpdc ligand (d).
2. **Figure S2.** TEM image of SiO_2@mTiO_2.
3. **Figure S3.** EDS mapping for each element in SiO_2@mTiO_2.
4. **Figure S4.** Luminescent lifetime of hybrid material Eu(tta)$_3$bpdc-SiO_2@mTiO_2 in state solid.
5. **Figure S5.** Excitation and emission spectra of hybrid material Eu(tta)$_3$bpdc-SiO_2@mTiO_2 in ethanol solution at room temperature.
6. **Figure S6.** Luminescent intensities of hybrid material Eu(tta)$_3$bpdc-SiO_2@mTiO_2 at 396 nm in presence of other metal ions and Al^{3+} ion in ethanol solution.
7. **Figure S7.** C 1s spectra for hybrid materials Eu(tta)$_3$bpdc-SiO_2@mTiO_2 and Eu(tta)$_3$bpdc-SiO_2@mTiO_2 + Al^{3+}.
8. **Figure S8.** The PXRD patterns of hybrid materials Eu(tta)$_3$bpdc-SiO_2@mTiO_2 and Eu(tta)$_3$bpdc-SiO_2@mTiO_2 + Al^{3+}.
9. **Figure S9.** TEM image of Eu(tta)$_3$bpdc-SiO_2@mTiO_2 + Al^{3+}.
10. **Figure S10.** EDS mapping for each element in Eu(tta)$_3$bpdc-SiO_2@mTiO_2 + Al^{3+}.
11. **Table S1.** The FT-IR characteristic bands of SiO_2@mTiO_2, SiO_2@mTiO_2-bpdc, Eu(tta)$_3$bpdc-SiO_2@mTiO_2 and H_2bpdc ligand.
12. **Table S2.** The characteristic diffraction peaks for PXRD in SiO_2@mTiO_2, SiO_2@mTiO_2-bpdc, Eu(tta)$_3$bpdc-SiO_2@mTiO_2 and Eu(tta)$_3$bpdc-SiO_2@mTiO_2 + Al^{3+}.
13. **Table S3.** CIE coordinates and emission colors of hybrid material Eu(tta)$_3$bpdc-SiO_2@mTiO_2 in different concentrations of Al^{3+} ion.
14. **Table S4.** Binding energies for XPS in hybrid materials Eu(tta)$_3$bpdc-SiO_2@mTiO_2 and Eu(tta)$_3$bpdc-SiO_2@mTiO_2+Al^{3+}.
15. **Table S5.** The mass ratio of each element in Eu(tta)$_3$bpdc-SiO_2@mTiO_2 and Eu(tta)$_3$bpdc-SiO_2@mTiO_2 + Al^{3+}.

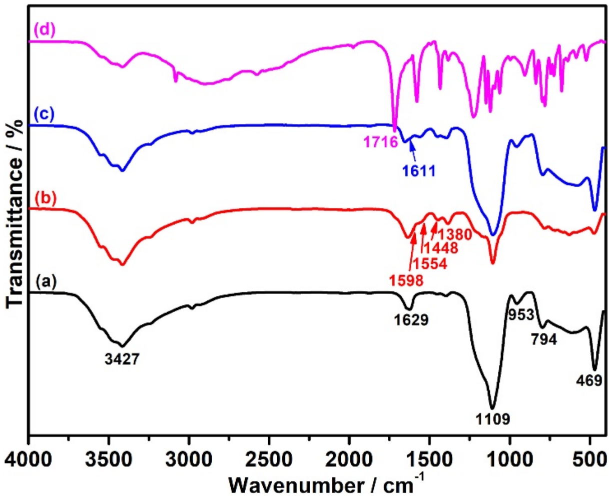

Figure S1. FT-IR spectra of SiO_2@mTiO_2 (a), SiO_2@mTiO_2-bpdc (b), Eu(tta)$_3$bpdc-SiO_2@mTiO_2 (c) and H_2bpdc ligand (d).

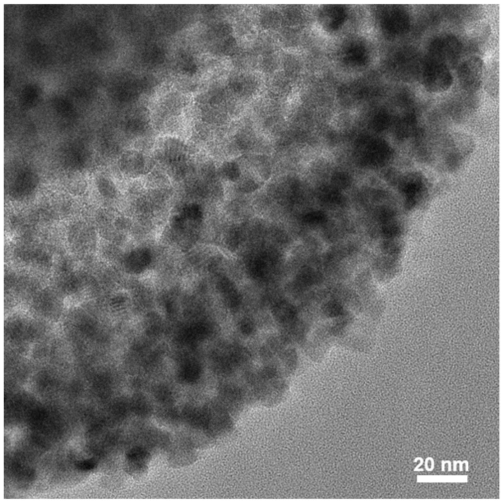

Figure S2. TEM image of SiO_2@mTiO_2.

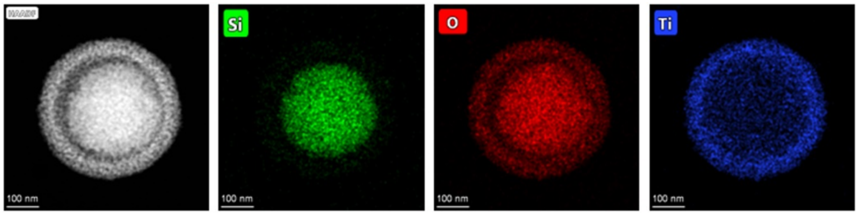

Figure S3. EDS mapping for each element in SiO_2@mTiO_2.

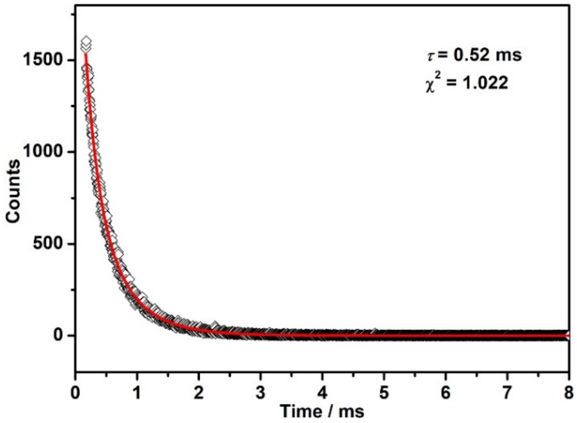

Figure S4. Luminescent lifetime of hybrid material Eu(tta)$_3$bpdc-SiO$_2$@mTiO$_2$ in state solid.

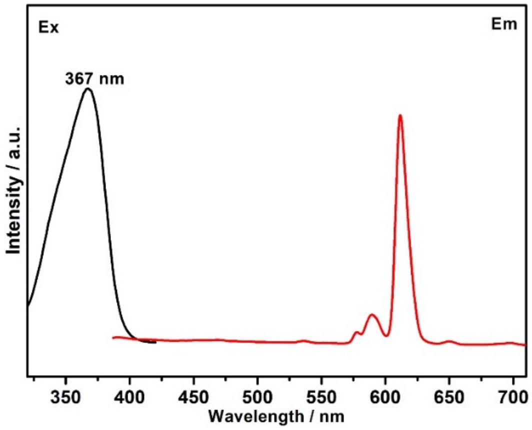

Figure S5. Excitation and emission spectra of hybrid material Eu(tta)$_3$bpdc-SiO$_2$@mTiO$_2$ in ethanol solution at room temperature.

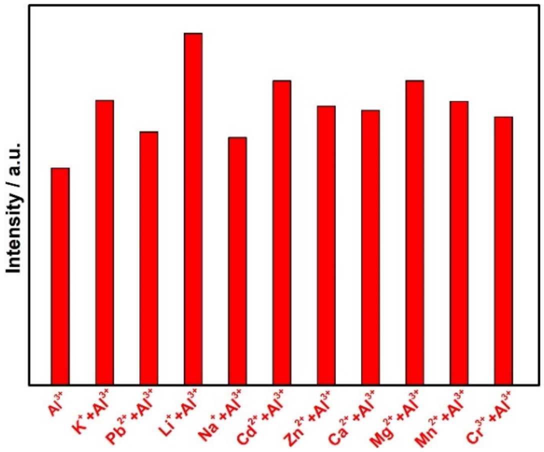

Figure S6. Luminescent intensities of hybrid material Eu(tta)$_3$bpdc-SiO$_2$@mTiO$_2$ at 396 nm in presence of other metal ions and Al^{3+} ion in ethanol solution.

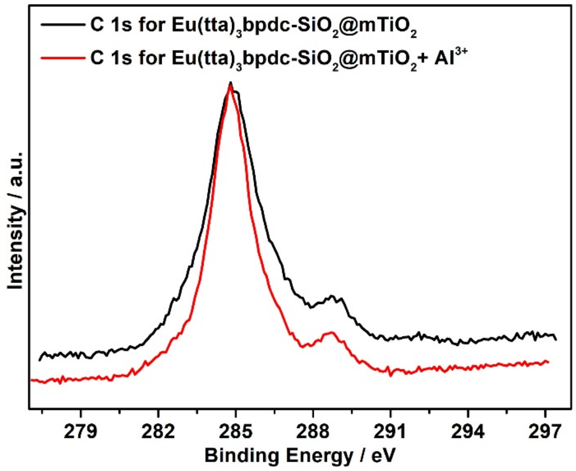

Figure S7. C 1s spectra for hybrid materials Eu(tta)$_3$bpdc-SiO_2@mTiO_2 and Eu(tta)$_3$bpdc-SiO_2@mTiO_2 + Al^{3+}.

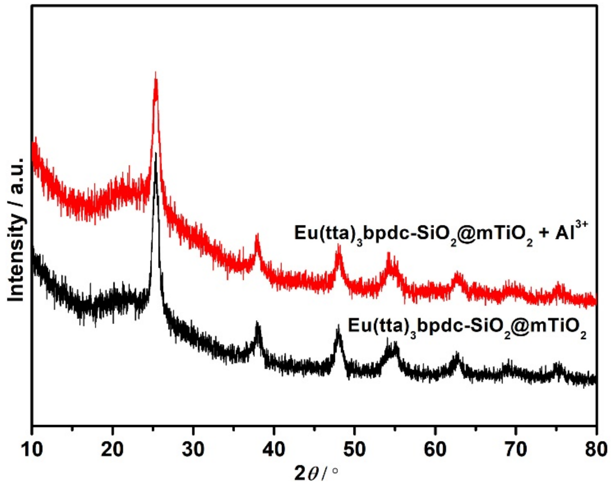

Figure S8. The PXRD patterns of hybrid materials Eu(tta)$_3$bpdc-SiO_2@mTiO_2 and Eu(tta)$_3$bpdc-SiO_2@mTiO_2 + Al^{3+}.

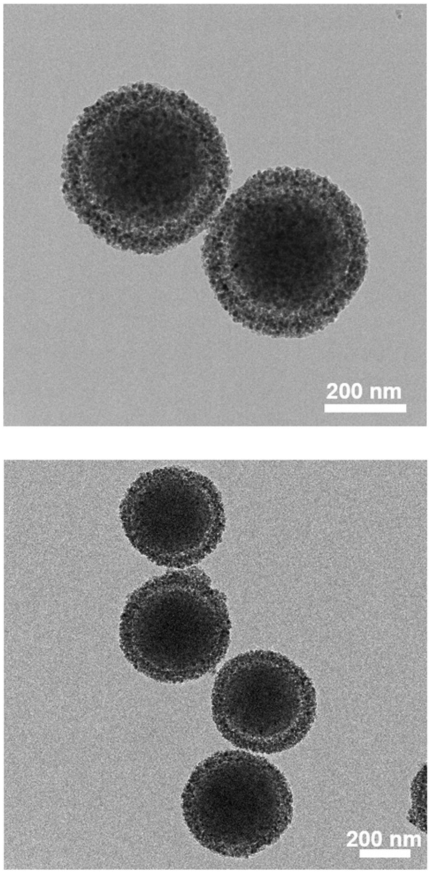

Figure S9. TEM image of Eu(tta)$_3$bpdc-SiO_2@mTiO_2 + Al^{3+}.

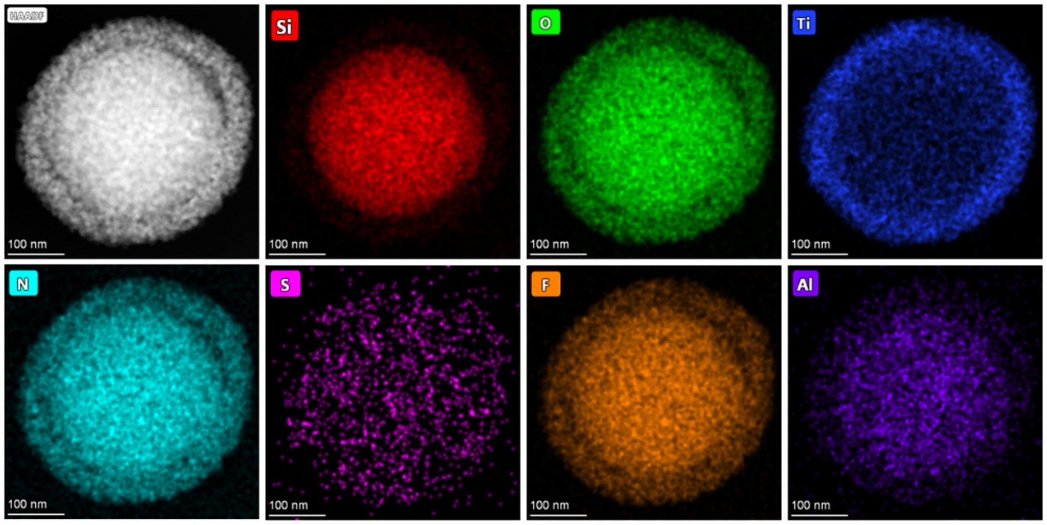

Figure S10. EDS mapping for each element in Eu(tta)$_3$bpdc-SiO_2@mTiO_2 + Al^{3+}.

Table S1. The FT-IR characteristic bands of SiO_2@mTiO_2, SiO_2@mTiO_2-bpdc, Eu(tta)$_3$bpdc-SiO_2@mTiO_2 and H_2bpdc ligand.

Characteristic Bands	Wavenumber / cm^{-1}	Ref.
Ti–O	450~750	[1]
Si–O asymmetric stretching vibration	1109	[2,3]
Si–O symmetric stretching vibration	794	[2,3]
Si–OH stretching vibration	953	[2,3]
carboxylic groups of H_2bpdc	1716	[1]
$\nu_{asym}(COO^-)$ of SiO_2@mTiO_2-bpdc	1554	[1,4–8]
$\nu_{sym}(COO^-)$ of SiO_2@mTiO_2-bpdc	1380	[1,4–8]
C=N of SiO_2@mTiO_2-bpdc	1598	[1]
C=N of Eu(tta)$_3$bpdc-SiO_2@mTiO_2	1611	[1]

Table S2. The characteristic diffraction peaks for PXRD in SiO_2@mTiO_2, SiO_2@mTiO_2-bpdc, Eu(tta)$_3$bpdc-SiO_2@mTiO_2 and Eu(tta)$_3$bpdc-SiO_2@mTiO_2 + Al^{3+}.

	SiO_2@mTiO_2	SiO_2@mTiO_2-bpdc	Eu(tta)$_3$bpdc-SiO_2@mTiO_2	Eu(tta)$_3$bpdc-SiO_2@mTiO_2 + Al^{3+}
(101)	25.27°	25.30°	25.33°	25.31°
(004)	37.84°	37.84°	37.84°	37.81°
(200)	48.03°	48.01°	48.01°	48.10°
(105)	54.23°	54.29°	54.30°	54.31°
(211)	55.04°	55.03°	55.03°	55.05°
(204)	62.64°	62.63°	62.62°	62.61°
(116)	69.32°	69.33°	62.34°	62.34°
(220)	71.29°	71.29°	71.29°	71.29°
(215)	75.63°	75.66°	75.66°	75.66°

Table S3. CIE coordinates and emission colors of hybrid material $Eu(tta)_3bpdc\text{-}SiO_2@mTiO_2$ in different concentrations of Al^{3+} ion.

Concentration of Al^{3+} ion (μM)	CIE (x, y)	Color
0	(0.565, 0.325)	Red
16	(0.454, 0.279)	Pink
33	(0.356, 0.231)	Pink
49	(0.303, 0.199)	Pink
66	(0.269, 0.177)	Pink
82	(0.245, 0.162)	Purplish pink
99	(0.227, 0.148)	Purplish pink
115	(0.210, 0.133)	Purplish pink
147	(0.193, 0.116)	Purplish blue
180	(0.183, 0.105)	Purplish blue
212	(0.177, 0.097)	Purplish blue
243	(0.175, 0.094)	Blue

Table S4. Binding energies for XPS in hybrid materials $Eu(tta)_3bpdc\text{-}SiO_2@mTiO_2$ and $Eu(tta)_3bpdc\text{-}SiO_2@mTiO_2 + Al^{3+}$.

Peak	$Eu(tta)_3bpdc\text{-}SiO_2@mTiO_2$	$Eu(tta)_3bpdc\text{-}SiO_2@mTiO_2\text{+}Al^{3+}$
C 1s	284.8	284.8
O 1s	529.8	529.8
N 1s	398.8	398.8
F 1s	687.8	685.8
Si 2p	102.8	102.8
Ti 2p	458.8	457.8
S 2p	164.8	164.8
Eu 3d	1138.4	------
Al 2p	------	74.8

Table S5. The mass ratio of each element in $Eu(tta)_3bpdc\text{-}SiO_2@mTiO_2$ and $Eu(tta)_3bpdc\text{-}SiO_2@mTiO_2 + Al^{3+}$.

Elements	Line	Weight / %	
		**$Eu(tta)_3bpdc\text{-}SiO_2@mTiO_2$**	**$Eu(tta)_3bpdc\text{-}SiO_2@mTiO_2 + Al^{3+}$**
Si	Kα	22.00	21.35
O	Kα	44.79	43.73
Ti	Kα	18.12	17.10
C	Kα	7.16	7.04
N	Kα	4.64	4.38
F	Kα	0.24	0.17
Eu	Lα	3.05	0.32
Al	Lα	----	5.91

References

1. Sun, L.; Wang, Z.; Zhang, J.Z.; Feng, J.; Liu, J.; Zhao, Y.; Shi, L. Visible and near-infrared luminescent mesoporous titania microspheres functionalized with lanthanide complexes: microstructure and luminescence with visible excitation. RSC Adv. 2014, 4, 28481–28489.
2. Zhang, Z.; Li, H.; Li, Y.; Yu, X. Full-color emission of a Eu^{3+}-based mesoporous hybrid material modulated by Zn^{2+} ions: Emission color changes for Zn^{2+} sensing via an ion exchange approach. *Dalton Trans*. **2019**, *48*, 10547–10556.
3. Li, H.; Li, Y.; Zhang, Z.; Pang, X.; Yu, X. Highly selective luminescent sensing of Cu^{2+} in aqueous solution based on a Eu(III)-centered periodic mesoporous organosilicas hybrid. *Mater. Design* **2019**, *172*, 107712.
4. Liu, P.; Li, H.R.; Wang, Y.G.; Liu, B.Y.; Zhang, W.J.; Wang, Y.J.; Yan, W.D.; Zhang, H.J.; Schubert, U. Europium complexes immobilization on titania via chemical modification of titanium alkoxide. *J. Mater. Chem.* **2008**, *18*, 735–737.
5. Nazeeruddin, M.K.; Humphry-Baker, R.; Liska, P.; Grätzel, M. Investigation of Sensitizer Adsorption and the Influence of Protons on Current and Voltage of a Dye-Sensitized Nanocrystalline TiO_2 Solar Cell. *J. Phys. Chem. B* **2003**, *107*, 8981–8987.
6. Nakamoto, K. *Infrared and Raman Spectra of Inorganic and Coordination Compounds*; John Wiley and Sons: Hoboken, NJ, USA, 1978.
7. Bai, C.; Wei, F.-H.; Hu, H.-M.; Yan, L.; Wang, X.; Xue, G.-L. New highly luminescent europium (III) complex covalently bonded with titania-based host via using a terpyridine carboxylate derivative linker for fluorescence sensing. *J. Lumin.* **2020**, *227*, 117545.
8. Wei, F.; Bai, C.; Hu, H.-M.; He, S.; Wang, X.; Xue, G. Novel luminescent europium-centered hybrid material covalently grafted with organically modified titania via 2-substituted imidazophenanthroline for fluorescence sensing. *J. Rare Earth* **2021**, *39*, 666–673.
